# Trajectories of care dependency and predictors following laparoscopic radical gastrectomy for gastric cancer: a longitudinal study

**DOI:** 10.3389/fonc.2026.1799607

**Published:** 2026-06-04

**Authors:** Zheng-Ke-Ke Tan, Wen-Zhen Tang, Huan-Huan Wen, Jin-Yan Yi, Bo-Hua Zhong, Qiu-Yan Lang

**Affiliations:** The First Affiliated Hospital of Guangxi Medical University, Nanning, China

**Keywords:** care dependence, gastric cancer, laparoscopic radical gastrectomy for gastric cancer, latent growth mixture modeling, trajectory

## Abstract

**Objective:**

To explore the potential categories of the trajectory of care dependency in patients with gastric cancer (GC) after laparoscopic radical gastrectomy. In addition, sociodemographic and clinical variables of each trajectory class were analyzed.

**Methods:**

This longitudinal observational study included 223 patients who underwent laparoscopic radical gastrectomy for GC. Care dependency (CD) was assessed using the Care Dependency Scale (CDS) at five postoperative time points: postoperative day 1 (T1), postoperative day 3 (T2), postoperative day 6 (T3), postoperative day 30 (T4) and 3 months (T5). SPSS and Mplus were used for statistical analysis. Latent growth mixture modeling (LGMM) was employed to analyze the longitudinal trajectories of care dependency from T1 to T5 in patients with GC. Furthermore, logistic regression was used to compare the sociodemographic and clinical characteristics of patients across the identified care dependency trajectory groups.

**Results:**

The care dependency trajectories of 206 patients with GC were classified into four potential categories, C1 (low dependency-stable group, 5.82%), C2 (high dependency-increased group, 22.33%), C3 (complete dependency-increased group, 62.13%), and C4 (persistent dependency group, 9.71%). Psychological resilience, age, social information support, time to first ambulation, BMI, and albumin were the primary predictor of the care dependency trajectory subtypes of patients with GC.

**Conclusion:**

There was heterogeneity in the care dependency trajectories of patients after laparoscopic radical gastrectomy. Nursing staff should effectively identify and pay attention to the trajectory types inconsistent with the overall trend and their influencing factors, and reduce the care dependency level of patients through intervention.

## 1 Introduction

According to the GLOBOCAN 2022 estimates, gastric cancer (GC) was the fifth most common cancer and the fifth leading cause of cancer death worldwide, with an estimated 968,000 new cases and 660,000 deaths in that year (1). In the same year, China accounted for 37.0% (358,000 new cases) of global GC diagnoses and 39.5% (260,000 deaths) of related fatalities, making it the third leading cause of cancer-related death in the country (2, 3). Both the incidence and mortality rates of GC exhibit a clear age-dependent pattern, where the number of cases and deaths rises with increasing age. The growth rate accelerates notably in individuals aged 40 and above, peaking among those aged 80 and over (4). The overall disease burden remains substantial.

With the increasing implementation of screening programs for high-risk populations, GC is now more frequently detected at an early stage. The management of GC involves a multimodal approach, including surgical resection, chemotherapy, radiotherapy, and endoscopic treatment. The primary treatment modality is surgical resection, which involves removal of the tumor and adjacent gastric tissue along with regional lymph node dissection, with the goal of achieving a curative outcome (5). Compared with conventional open surgery, laparoscopic techniques offer distinct advantages, including reduced blood loss, a lower incidence of complications, and a faster recovery period. Given these benefits, they have been extensively integrated into clinical use (5, 6). However, the stress response induced by anesthesia and surgery often causes patients to experience short-term symptoms including pain, fatigue, and restricted mobility, leading to a marked deterioration in both physical function and quality of life. This decline consequently creates a need for reliance on others to complete daily activities.With the advancement of Enhanced Recovery After Surgery (ERAS) protocols, the hospital stay for GC patients has been reduced to 6–9 days (7, 8). Achieving readiness for discharge within this shortened timeframe requires patients to adapt to substantial changes across physiological, psychological, and social dimensions.

Dependency refers to a complex and integrated state in which an individual’s physical functioning is compromised, thereby requiring assistance from others (9). Care Dependency refers to a formal, professional care relationship wherein patients with diminished self-care ability or heightened care needs receive compensatory assistance from nurses or caregivers to restore their autonomy (10). In this study, the level of care dependency was quantified and assessed using the CDS scale. The scale was developed based on Virginia Henderson’s nursing theory, and it includes not only functional limitations but also psychological dependence on caregivers (11, 12).

Nursiswati et al. (13) reported that the functional status of cancer patients declined significantly within the first year after diagnosis, a change which may be associated with the surgical treatment they subsequently underwent. Okuyama et al. (14) demonstrated that postoperative patients with GC experience a progressive decline in their activities of daily living (ADL). Research indicates that the postoperative recovery process involves substantial changes in physical function and disease adaptation, resulting in a dynamic trajectory of care dependency (15). The amplitude of this change is reported to be significantly greater than that observed in patient groups with non-surgical primary conditions, such as stroke or chronic heart failure (16, 17).

Most previous studies have assessed care dependency only at single time points or assumed a homogeneous recovery pattern across all patients, thereby overlooking potential heterogeneity in postoperative trajectories (14, 18). To address this unresolved issue, the present study employed Latent Growth Mixture Modeling (LGMM), a method that identifies distinct latent subpopulations with different change trajectories (19, 20), to delineate the patterns of care dependency after laparoscopic radical gastrectomy for gastric cancer and to explore their determinants.

## 2 Materials and methods

### 2.1 Design

This longitudinal descriptive study investigated the experiences of GC patients. Participants were recruited from the gastrointestinal surgery department of a tertiary hospital in Guangxi, China between September 2023 and November 2024. Ethical approval for this study was obtained from The First Affiliated Hospital of Guangxi Medical University Human Research Ethics Committee (2024-K0463). This observational study followed the STROBE guidelines in Supplementary File A.

All patients followed a standardized ERAS protocol for GC, which included early ambulation, pulmonary rehabilitation training, early removal of the nasogastric tube and surgical drains. All patients routinely received 3−4 abdominal drains. Drains were removed when the daily output was consistently below 50 mL for 2−3 consecutive days and the attending physician confirmed no clinical or biochemical signs of leakage or infection. A graded feeding schedule was implemented, and oral nutritional supplements (ONS) were provided when oral intake was insufficient. After discharge, patients were advised to continue ONS (at least 400 kcal per day) for four weeks in addition to normal food intake, to maintain regular physical activity, and to attend outpatient follow−up at day 30 and month 3 after discharge. All patients received the same written discharge instructions.

### 2.2 Participants

A convenience sampling method was employed. The inclusion criteria for patients were as follows (1): Diagnosis consistent with the Chinese Society of Clinical Oncology (CSCO) Gastric Cancer Diagnosis and Treatment Guidelines (2023) (21). (2) Age≥18 years; (3) Successful laparoscopic standard radical gastrectomy for GC (R0 resection); (4) Clear consciousness with adequate comprehension and communication abilities. The exclusion criteria were as follows: (1) Other active malignancies; (2) Severe cardiopulmonary/organ dysfunction; (3) Postoperative complications of Clavien-Dindo grades III-V; (4) Eastern Cooperative Oncology Group (ECOG) score < 3 (indicating independence in daily activities prior to surgery, as patients with scores ≥ 3 already have substantial care dependency that could confound the longitudinal analysis); (5) Postop readmission.

Withdrawal Criteria: (1) Patient withdrawal; (2) Death; (3) Clinical deterioration preventing participation; (4) Lost to follow-up.

Sample size estimation, based on a table for single-group repeated measures (22) (5 measurements, r=0.5, f=0.14, α=0.05, power=0.8) with a 20% attrition buffer, indicated a minimum of 152 patients. Research indicates that when using the Bayesian Information Criterion as the primary consideration for model selection, to ensure model identification accuracy for latent growth modeling (23), the target was set to ≥200.

### 2.3 Measures

#### 2.3.1 Demographic and clinical characteristics questionnaire

A researcher-designed questionnaire, based on a literature review, was used to collect demographic and clinical data. It included: gender, age, educational level, type of health insurance, marital status, place of residence, primary caregiver, number of comorbid chronic conditions, tumor stage, surgical approach, chemotherapy status and regimen, 6-minute walk distance, NRS2002 score, PG-SGA score, duration of surgery, Body Mass Index (BMI), preoperative albumin level, preoperative total lymphocyte count, time to first ambulation and postoperative length of hospital stay (LOS).

#### 2.3.2 Care dependency scale

The Care Dependency Scale was originally developed by Dijkstra et al. (12) and later translated and cross-culturally adapted into Chinese by Zhang Shuqi et al. (24). This 15-item scale assesses dependency across physiological, psychological, and social domains, addressing the limitation of traditional ADL scales which focus solely on physical function. It employs a 5-point Likert scale, with a total score ranging from 15 to 75. It is a reverse-scored instrument, where a lower score indicates a higher level of care dependency. The score is interpreted as follows: 15-24 (complete dependency), 25-44 (very high dependency), 45-59 (partial dependency), 60-69 (low dependency), and ≥70 (independent). The scale has demonstrated good reliability and validity, with a reported Cronbach’s α coefficient of 0.944 in a cancer patient population (25).

#### 2.3.3 Patient-reported outcomes measurement information system social support short form

The PROMIS measurement system was developed under the auspices of the U.S. National Institutes of Health (NIH) and was cross-culturally adapted into Chinese by Wu Fulei (26). This short form comprises 12 items across three dimensions: instrumental support, emotional support, and informational support. Items are rated on a 5-point Likert scale from “never” to “always,” with each dimension score ranging from 12 to 60. A higher score indicates a greater perceived level of social support. The Cronbach’s α coefficients for all dimensions have been reported to exceed 0.90.

#### 2.3.4 10-Item Connor-Davidson resilience scale

The 10-item Connor-Davidson Resilience Scale, compiled by Campbell-Sills et al. (27), was used in its Chinese version adapted by Wang et al. (28) to assess patients’ psychological resilience. The scale consists of 10 items, each rated on a 5-point Likert scale from “almost never” to “almost always,” with scores from 1 to 5.

#### 2.3.5 International physical activity questionnaire short form

The IPAQ Short Form, translated and adapted into Chinese by Qu Ningning et al. (29), was used to assess sedentary behavior and physical activity in different contexts over the past 7 days. The questionnaire contains 7 items covering four dimensions: sedentary time, vigorous-intensity physical activity, moderate-intensity physical activity, and walking. It has shown good reliability with a Cronbach’s α coefficient of 0.826.

### 2.4 Statistical analysis

To identify distinct trajectories of care dependency over time, we used LGMM. LGMM conceptualizes the population as composed of multiple unobserved subpopulations (latent classes), each with its own growth trajectory (intercept and slope). It allows probabilistic classification of individuals into classes and estimates class−specific trajectory parameters. LGMM was performed using Mplus 8.0 software. Care dependency scores from the five measurement time points (T1-T5) were used to fit the models. The analysis began with a single-class (unconditional growth) model, and the number of latent classes was incrementally increased. The optimal model was selected by comparing model fit indices.

The following fit indices were used to evaluate and compare competing models:

Information Criteria: Akaike Information Criterion (AIC), Bayesian Information Criterion (BIC), and sample-size adjusted BIC (aBIC). Lower values on these indices indicate a better model fit.Classification Accuracy: Entropy, a metric ranging from 0 to 1, where values closer to 1 indicate more precise classification of individuals into latent classes. Comparative Fit Tests: The Lo-Mendell-Rubin Likelihood Ratio Test (LMR-LRT) and the Bootstrap Likelihood Ratio Test (BLRT). Significant P-values (*P* < 0.05) for these tests suggest that a model with “k” classes fits the data significantly better than a model with “k-1” classes.

All other statistical analyses were conducted using SPSS software. Continuous variables are presented as mean ± standard deviation and were compared using analysis of variance (ANOVA). Categorical variables are presented as number (percentage) and were compared using the Chi-square test or Fisher’s exact test, as appropriate. The identified latent class membership was subsequently treated as the dependent variable. Univariate logistic regression analyses were first performed to examine individual associations between predictor variables and class membership. Variables showing a significant association (*P* < 0.05) in the univariate analysis were then entered into a multivariate logistic regression model to identify independent predictors. A two-tailed P-value of less than 0.05 was considered statistically significant.

### Data collection and time points

(1) Baseline data (T0) were collected one day before surgery.

①The following information was extracted from the hospital’s electronic medical record system: age, medical history, preoperative laboratory values including albumin, and other relevant clinical variables. ②The PROMIS Social Support, CD-RISC, and IPAQ-SF scales were assessed using self-report questionnaires completed by the patients. ③Nutritional status was assessed by specialized nutrition nurses using the PG−SGA and NRS2002 before surgery (T0).

(2) A longitudinal design was implemented to track care dependency.

Based on key timepoints in the ERAS perioperative management pathway for gastrointestinal cancer (30) and clinical feasibility, five postoperative follow-up assessments were scheduled: postoperative day 1 (T1), day 3 (T2), day 6 (T3), day 30 (T4), and month 3 (T5). These time points were chosen because T1 to T3 capture the rapid early changes during the acute ERAS milestones (ambulation, oral intake, drain removal), T4 reflects the hospital−to−home transition, and T5 represents the mid−term stable recovery phase. This schedule balances clinical relevance, statistical power for trajectory modeling, and feasibility in routine practice. At each of these time points, the Chinese version of the Care Dependency Scale was administered.

The CDS, an observer−rated scale, was administered by uniformly trained researchers. The assessment method was based on direct observation or brief interview with the patient. Postoperative CDS assessments were scheduled at five time points: T1, T2, T3, T4, and T5. During hospitalization (T1–T3), all assessments were conducted face−to−face. After discharge (T4–T5), face−to−face follow−up was the primary mode, supplemented by telephone or online interviews when an in−person visit was not feasible.

### 2.6 Quality control measures

Multiple strategies were employed to ensure data quality and participant retention:

#### 2.6.1 Standardized administration

Before surgery, patients were encouraged to complete the scales independently. For those unable to do so, investigators read each item aloud using simple, non-technical language and recorded the responses based on the patient’s answers. The researchers received standardized training before conducting the CDS assessments.

#### 2.6.2 Participant Retention

To minimize loss to follow-up after discharge, contact information for patients, their primary caregivers, or family members was securely retained. Prior to each follow-up assessment, patients were contacted to schedule a convenient time.

#### 2.6.3 Flexible follow-up

Post-discharge surveys (T4, T5) were conducted using a combination of methods to maximize response rates, including face-to-face interviews during scheduled clinical visits, telephone calls, and online.

## 3 Results

### 3.1 General characteristics of the gastric cancer patients

**Figure 1:**
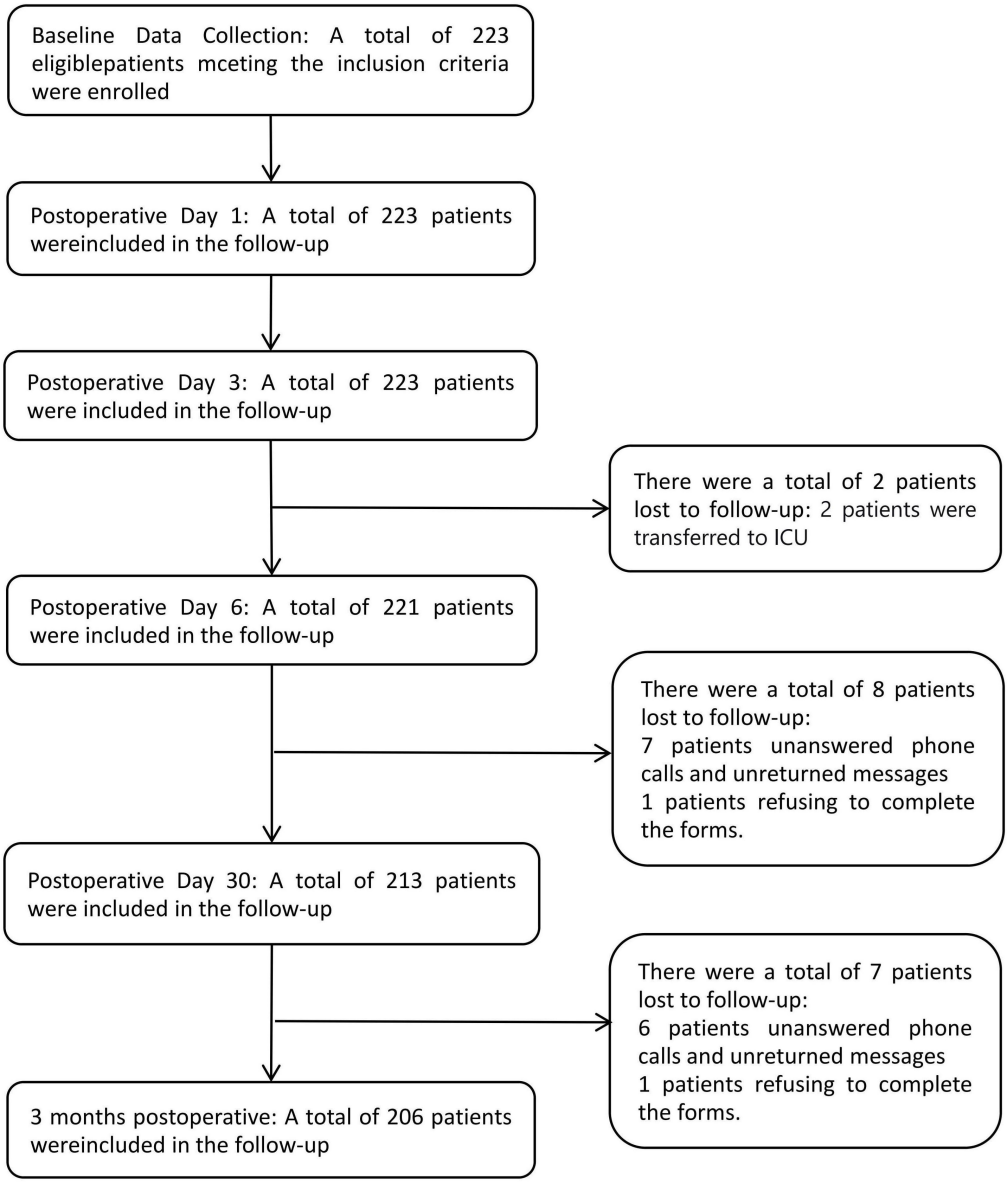
Participant flow diagram. The diagram shows the number of patients assessed for eligibility, excluded, and finally included in the analysis. Reasons for exclusion are provided in the boxes.

In the baseline data survey, a total of 223 patients were enrolled, with 206 ultimately completing all five surveys. The overall attrition rate was 7.62% (17/223), with reasons for loss to follow-up including transfer to ICU (n=2), failure to respond to phone calls (n=13), and voluntary withdrawal (n=2). The follow-up process is illustrated in **Figure 1**. The mean age of the final cohort (n=206) that completed all follow-ups was 58.57 ± 0.78 years, with a range from 19 to 86 years. No patient in our cohort required additional organ resection. Other demographic and clinical characteristics are presented in **Table 1**.

**Table 1:** General characteristics of the gastric cancer patients.

Factors	Inclusion of 206 patients	
	n/Mean	%/SD
Age	58.57	0.78
Gender		
Male	147	71.36
Female	59	28.64
Educational Level		
Junior high school or below	129	62.62
Senior high school or secondary specialized school	47	22.82
College or above	30	14.57
Occupation		
Clerk	33	16.02
Farmer	122	59.22
Unemployed	17	8.25
Retired	34	16.50
Type of Health Insurance		
Employee Medical Insurance	48	23.30
Resident Medical Insurance	153	74.27
Out-of-pocket/Self-pay	5	2.43
Marital Status		
Married	191	92.72
Unmarried/Divorced/Widowed	15	7.28
Residence		
Urban	54	26.21
Rural	111	53.88
Town	41	19.90
Primary Caregiver		
Children	105	50.97
Spouse	71	34.47
Other	30	14.56
Tumor Stage		
Stage I	41	19.90
Stage II	76	36.89
Stage III	82	39.81
Stage IV	7	3.40
Number of Comorbidities		
0	47	22.82
1–3	90	43.69
>3	69	33.50
Surgical Procedure		
Total Gastrectomy	39	18.93
Distal Gastrectomy	156	75.73
Proximal Gastrectomy	11	5.34
Lymphadenectomy		
D1	14	6.80
D2 or D2+	192	93.20

### 3.2 Postoperative care dependency scores at five time points

The care dependency scores of patients after laparoscopic radical gastrectomy at time points T1-T5 were (26.71 ± 13.72), (45.08 ± 10.68), (56.75 ± 9.10), (71.90 ± 4.14), and (72.53 ± 3.23), respectively, showing an overall upward trend. A repeated-measures analysis of variance (ANOVA) revealed a statistically significant difference in care dependency scores across the five time points (F = 931.91, P < 0.001). Multiple comparison results indicated that T5 = T4 > T3 > T2 > T1. The scores for each item at the five time points are detailed in Supplementary File B.

### 3.3 Model fit indices of growth mixture models for care dependency in patients following laparoscopic radical gastrectomy

The LGMM fit indices suggested that the four trajectories model was optimal. It exhibited the lowest values for AIC, BIC, and aBIC, an Entropy value of 0.955, and statistically significant results for both the LMR and BLRT tests. Considering both statistical indices and clinical interpretability, the four-class solution was retained. The detailed model fit statistics are presented in **Table 2**.

**Table 2:** Trajectories of care dependency in patients following laparoscopic radical gastrectomy: growth mixture model.

Classes	LL	AIC	BIC	aBIC	Entropy	*P*		Proportion
						LMR	BLRT	
1	-3287.548	6601.097	6644.359	6603.170	–	–	–	–
2	-3231.079	6494.158	6547.404	6496.710	0.956	0.0423	<0.001	0.718/0.281
3	-3189.273	6416.545	6479.775	6419.575	0.947	0.4290	<0.001	0.272/0.631/0.097
4	-3155.034	6354.067	6427.280	6357.576	0.955	0.0405	<0.001	0.223/0.621/0.097/0.058
5	-3145.683	6341.366	6424.563	6345.353	0.948	0.690	<0.001	0.112/0.558/0.004/0.277/0.049

AIC, Akaike Information Criterion; BIC, Bayesian Information Criterion; aBIC, sample-size adjusted Bayesian Information Criterion; LMR, Lo-Mendell-Rubin likelihood ratio test; BLRT, Bootstrap Likelihood Ratio Test.

### 3.4 Trajectory of care dependency in patients following laparoscopic radical gastrectomy

**Figure 2:**
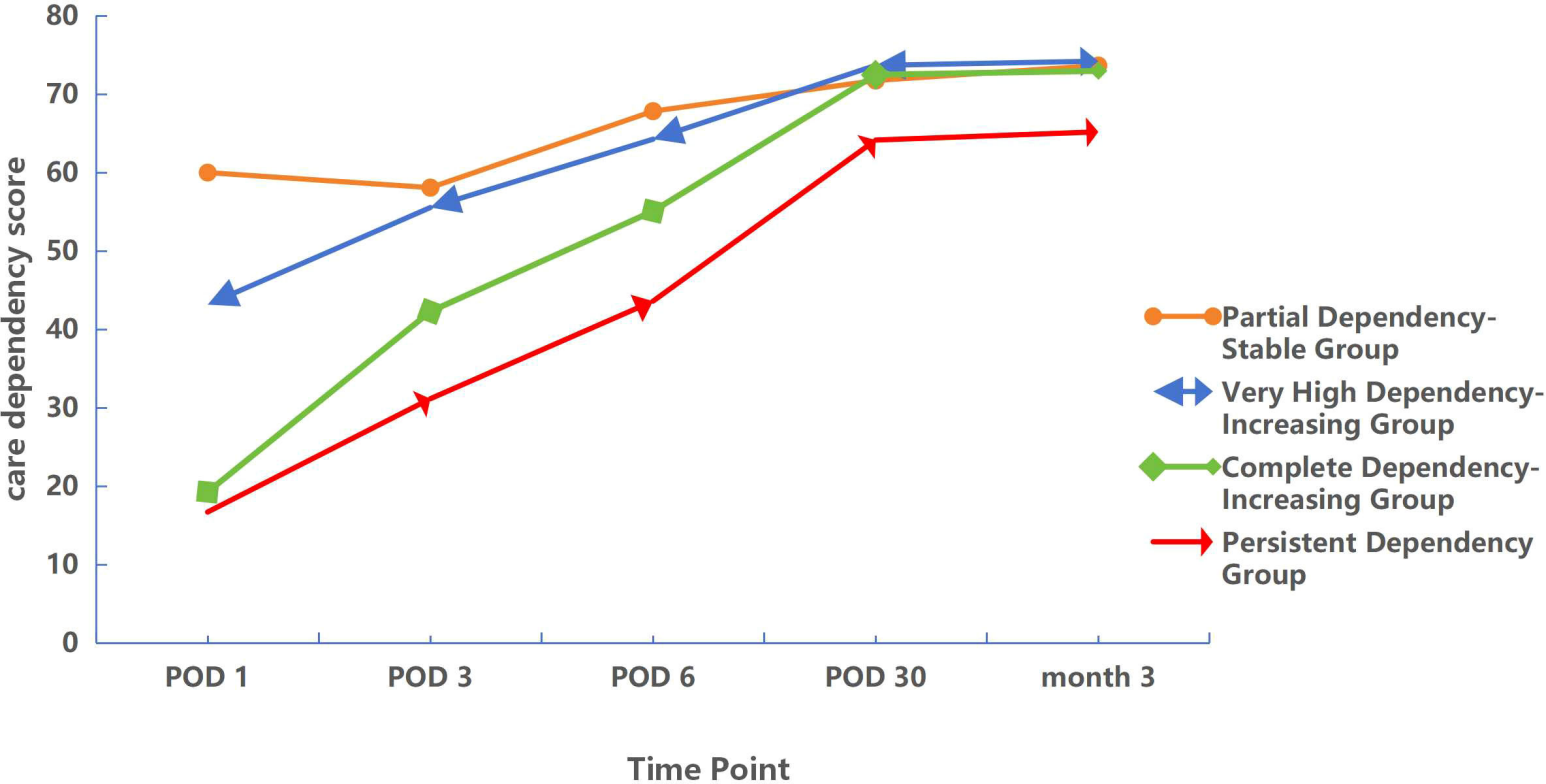
Trajectories of care dependency in patients with gastric cancer. The x-axis shows the five assessment time points. The y-axis represents the total CDS score, with higher scores indicating lower care dependency. Each trajectory class is labeled on the figure.

The trajectories of the four latent classes are plotted in **Figure 2**, with mean care dependency scores on the vertical axis and assessment time points on the horizontal axis. Each class was named based on the characteristics of its score trajectory:

1. Class 1 (Partial Dependency-Stable Group): This group, comprising 5.82% (12/206) of the sample, started with a relatively high baseline score (intercept = 58.788), indicating low initial dependency, and showed the slowest rate of improvement (slope=5.946, *P* < 0.001).
2. Class 2 (Very High Dependency-Increasing Group): Accounting for 22.33% (46/206) of participants, this group had a moderate initial score (intercept = 43.156) and exhibited a significant upward trend (slope=12.723, *P* < 0.05).
3. Class 3 (Complete Dependency-Increasing Group): This was the largest class (62.13%, 139/206). It started with the lowest baseline score (intercept=19.402), signifying the highest initial dependency, and demonstrated the most rapid rate of improvement (slope=21.952, *P* < 0.001). At T4 (day 30), the CDS score of the group was no longer significantly different from those of the Partial dependency and Very low dependency groups (*P* > 0.05).
4. Class 4 (Persistent Dependency Group): Representing 9.71% (20/206) of the sample, this group began with a very low score (intercept=15.966) and showed significant improvement (slope=20.188, *P* < 0.001). Notably, their scores remained below the independence threshold (70 points) at all five time points, indicating a state of sustained care dependency.

### 3.5 Univariate analysis of care dependency trajectories in patients following laparoscopic radical gastrectomy for gastric cancer

The univariate analysis revealed statistically significant differences among the four trajectory classes in the following variables: age, number of comorbidities, tumor stage, surgical approach, NRS 2002, PG-SGA, 6-minute walking distance, BMI, preoperative albumin level, time to first ambulation, social support score, and psychological resilience score. Detailed results are presented in **Table 3**.

**Table 3:** Results of univariate analysis of care dependency trajectories in patients following laparoscopic radical gastrectomy for gastric cancer (n=206).

Fators	Partial dependency-stable group(n=12)	Very High dependency-increasing group(n=46)	Complete dependency-increasing group(n=128)	persistent dependency group (n=20)	Statistic	*P*
Age					22.269^3)^	0.001
<45 Years	5(41.67)	7(15.22)	11(8.59)	0(0.00)		
>45 and ≤59 Years	3(25.00)	24(52.17)	48(37.50)	6(30.00)		
≥60 Years	4(33.33)	15(32.61)	69(53.91)	14(70.00)		
Gender					1.231^2)^	0.746
Male	10(83.33)	31(67.39)	92(71.88)	14(70.00)		
Female	2(16.67)	15(32.61)	36(28.13)	6(30.00)		
Educational Level					1.454^3)^	0.228
Junior high school or below	8(66.67)	32(69.57)	75(58.59)	14(70.00)		
Senior high school or secondary specialized school	4(33.33)	9(19.57)	32(25.00)	2(10.00)		
College or above	0(0.00)	5(10.87)	21(16.41)	4(20.00)		
Occupation					2.841^2)^	0.092
Clerk	2(16.67)	11(23.91)	19(14.84)	1(5.00)		
Farmer	7(58.33)	26(56.52)	76(59.38)	13(65.00)		
Unemployed	1(8.33)	5(10.87)	11(8.59)	0(0.00)		
Retired	2(16.67)	4(46.00)	22(17.19)	6(30.00)		
Health Insurance					0.540^2)^	0.462
Employee Medical Insurance	2(16.67)	9(19.57)	31(24.22)	6(30.00)		
Resident Medical Insurance	10(83.33)	36(78.26)	94(73.44)	13(65.00)		
Self-pay	0(0.00)	1(2.17)	3(2.34)	1(5.00)		
Marital Status					0.267^2)^	0.605
Married	10(83.33)	42(91.30)	122(95.31)	17(85.00)		
Unmarried/Divorced/Widowed	2(16.67)	4(8.70)	6(4.69)	3(15.00)		
Residence					0.015^2)^	0.902
Urban	1(8.33)	13(28.26)	34(26.56)	6(30.00)		
Rural	9(75.00)	27(58.70)	66(51.56)	9(45.00)		
Town	2(16.67)	6(13.04)	28(21.88)	5(25.00)		
Primary Caregiver					2.678^2)^	0.102
Children	4(33.33)	24(52.17)	61(47.66)	16(80.00)		
Spouse	4(33.33)	17(36.96)	48(37.50)	2(10.00)		
Other	4(33.33)	5(10.87)	19(14.84)	2(10.00)		
Number of Comorbidities					13.236^3)^	0.001
0	4(33.33)	16(34.78)	24(18.75)	3(15.00)		
1~3	7(58.33)	22(47.83)	55(42.97)	6(30.00)		
>3	1(12.00)	8(17.39)	49(38.28)	11(55.00)		
Tumor Stage					7.374^3)^	0.007
Stage I	4(33.33)	12(26.09)	23(17.97)	2(10.00)		
Stage II	5(41.67)	18(39.13)	51(39.84)	2(10.00)		
Stage III	2(16.67)	14(30.43)	51(39.84)	15(75.00)		
Stage IV	1(8.33)	2(4.35)	3(2.34)	75(14.29)		
Surgical Procedure					6.062^2)^	0.014
Total Gastrectomy	0(0.00)	2(4.35)	31(24.22)	6(30.00)		
Distal Gastrectomy	12(100.00)	42(91.30)	89(69.53)	13(65.00)		
Proximal Gastrectomy	0(0.00)	2(4.35)	8(6.25)	1(5.00)		
Lymphadenectomy						
D1	2(16.67)	6(13.04)	6(4.69)	0(0.00)	6.512^2)^	0.057
D2 or D2+	10(83.33)	40(86.96)	122(95.31)	20(100.00)		
NRS2002					17.899^2)^	<0.001
<3	10(83.33)	35(76.09)	78(60.94)	5(25.00)		
≥3	2(16.67)	11(23.91)	50(39.06)	15(75.00)		
PG-SGA					13.335^3)^	<0.001
0~1	3(25.00)	13(28.26)	19(14.84)	0(0.00)		
2~3	5(41.67)	15(32.61)	43(33.59)	4(20.00)		
4~8	2(16.67)	13(28.26)	42(32.81)	24(18.75)		
>9	2(16.67)	5(10.87)	24(18.75)	10(50.00)		
Duration of Surgery	283.42 ± 64.85	307.78 ± 90.73	321.85 ± 96.64	317.70 ± 73.27	0.801^1)^	0.494
6-Minute Walk Distance	505.02 ± 103.16	476.86 ± 86.93	440.99 ± 91.37	393.85 ± 85.4	5.785^1)^	0.001
BMI(kg/m²)	24.03 ± 1.18	23.23 ± 2.75	22.05 ± 2.98	19.45 ± 2.77	10.076^1)^	<0.001
Preoperative Albumin(g/L)	39.86 ± 2.60	38.71 ± 3.17	36.202 ± 3.98	35.78 ± 4.15	8.071^1)^	<0.001
Preoperative Total Lymphocyte Count(10^9^/L)	2.09 ± 0.55	1.92 ± 0.66	1.79 ± 0.47	1.94 ± 0.45	1.848^1)^	0.140
Time to First Ambulation (h)	21.08 ± 5.10	28.06 ± 12.23	42.22 ± 23.31	47.03 ± 22.48	9.336^1)^	<0.001
Postoperative LOS (d)	10.41 ± 1.28	8.39 ± 0.31	10.07 ± 0.26	11.60 ± 1.08	1.559^1)^	0.079
PROMIS Social Support	52.25 ± 8.89	49.43 ± 7.82	43.89 ± 7.90	42.65 ± 8.36	9.383^1)^	<0.001
Instrumental Support^4)^	18.75 ± 2.42	18.13 ± 2.51	16.87 ± 3.02	16.80 ± 2.80	3.480^1)^	0.017
Emotional Support^5)^	17.5 ± 3.40	16.78 ± 2.72	14.15 ± 2.68	13.40 ± 2.68	16.055^1)^	<0.001
Informational Support^6)^	16.00 ± 3.91	14.52 ± 4.20	12.99 ± 4.21	12.45 ± 4.47	3.333^1)^	0.020
CD-RISC	26.33 ± 7.58	31.60 ± 5.70	27.79 ± 7.74	24.45 ± 8.19	5.390^1)^	<0.001
IPAQ-SF (MET-min/d)	1557.61 ± 868.15	1538.48 ± 1089.87	1050.59 ± 785.31	1057.97 ± 653.05	4.564^1)^	0.004

1)Chi-square test; 2) χ² value; 3) Kruskal-Wallis value.

### 3.6 Logistic regression analysis of latent care dependency trajectory classes in patients following laparoscopic radical gastrectomy for gastric cancer

Using the four care dependency trajectory classes as the dependent variable with the “Partial Dependency-Stable Group” as the reference, logistic regression analysis was performed. All variables that showed significance in the univariate analysis were included as independent variables. The assignment of independent variables was as follows: continuous variables, including 6-minute walking distance, BMI, preoperative albumin, time to first ambulation, scores for each dimension of social support, psychological resilience score, and total physical activity level, were entered as their actual values. Age: <45 years = 1; 45–59 years = 2; ≥60 years = 3. Number of Comorbidities: 0 = 1; 1–3 = 2; >3 = 3. Tumor Stage: Stage I = 1; Stage II = 2; Stage III = 3; Stage IV = 4. Surgical Approach: Total gastrectomy (Z1 = 0, Z2 = 0); Distal gastrectomy (Z1 = 1, Z2 = 0); Proximal gastrectomy (Z1 = 0, Z2 = 1). NRS 2002 Score: <3 = 0; ≥3 = 1. PG-SGA Score: 0–1 = 1; 2–3 = 2; 4–8 = 3.

The logistic regression analysis identified the following factors as significant independent predictors of latent class membership: age, psychological resilience, the informational support dimension of social support, time to first ambulation, BMI, and low preoperative albumin level. The detailed results of the logistic regression are shown in **Table 4**.

**Table 4:** Logistic regression analysis of latent care dependency trajectory classes in postoperative gastric cancer patients.

Latent class	Affect variables	β	SE	Wald ϰ^2^	*P*	OR	95% CI
Very High Dependency-Increasing Group	CD-RISC	0.209	0.100	4.380	0.036	1.232	1.013~1.498
	Age						
	≤45 Years	0	–	–	–	–	–
	>45 and ≤59 Years	-4.813	2.284	4.442	0.035	0.008	0~0.714
Complete Dependency-Increasing Group	PROMIS Social Support-Informational Support	-0.752	0.321	5.466	0.019	0.471	0.251~0.886
	Time to First Ambulation(h)	0.228	0.106	4.642	0.031	1.256	1.021~1.545
	BMI(kg/m²)	-0.602	0.288	4.358	0.037	0.548	0.311~0.964
	preoperative albumin(g/L)	-0.856	0.292	8.606	0.003	0.425	0.240~0.753
	Age						
	≤45 Years	0	–	–	–	–	–
	>45 and ≤59 Years	-4.752	2.318	4.201	0.040	0.009	0~0.812
Persistent Dependency Group	PROMIS Social Support-Informational Support	-0.847	0.358	5.619	0.018	0.429	0.213~0.864
	Time to First Ambulation(h)	0.215	0.107	4.057	0.044	1.240	1.006~1.529
	BMI(kg/m²)	-0.909	0.317	8.248	0.004	0.403	0.217~0.749
	preoperative albumin(g/L)	-0.772	0.304	6.435	0.011	0.462	0.255~0.839

## 4 Discussion

In this study, care dependency levels of patients after laparoscopic radical gastrectomy for GC were assessed from postoperative day 1 to month 3. The results showed that CDS scores increased progressively at five postoperative time points, indicating a dynamic process of continuously decreasing care dependency. This finding is consistent with the study by Han et al. (31). The care dependency scores from postoperative day 1 to day 6 were below 70, which may be attributed to the trauma and stress induced by anesthesia and surgery, leading to reduced physical strength and mobility (32) and consequently a higher level of dependency. At postoperative day 30 and month 3, care dependency status stabilized, with scores exceeding 70, suggesting that patients who underwent laparoscopic radical gastrectomy regained self−care ability by day 30 and no longer required assistance. With the development of ERAS, the current average length of hospital stay is significantly shorter than the traditional postoperative recovery period, requiring patients to complete physical and psychological rehabilitation within a limited hospital stay (33).

From T1 to T5, our item level analysis showed that the most pronounced improvements were observed in physical-related items, including diet, mobility, and recreational activities. This finding suggests that patients experience greater changes in physical function during the postoperative recovery phase, particularly a higher level of physiological dependency in the early postoperative period. Healthcare providers should pay more attention to care dependency in the early postoperative phase, implement timely interventions to promote recovery, and reduce the burden on caregivers. Although communication and social contact showed only modest changes, these aspects may still negatively affect long-term quality of life and mental health. Therefore, future postoperative rehabilitation programs should not only focus on early mobilization and nutritional support but also incorporate targeted psychosocial interventions, such as communication training or peer support groups.

The LGMM analysis delineated four distinct latent classes of care dependency trajectories following laparoscopic radical gastrectomy, all of which demonstrated a dynamic declining trend, with “Partial Dependency-Stable Group” accounting for 5.82%, “Very High Dependency-Increasing Group” accounting for 22.33%, “Complete Dependency-Increasing Group” accounting for 62.13%, “Persistent Dependency Group” accounting for 9.71%. A significant majority of patients (71.8%) were classified into either the “Complete Dependency-Increasing Group” or the “Persistent Dependency Group.” This indicates that most patients initially experienced a high degree of dependency, a finding consistent with the research by Li et al. (34). While dependency gradually decreased with postoperative recovery for most, this pattern underscores the necessity for healthcare providers to enhance ward rounds and structured health education during the early postoperative period to support this critical recovery phase (35).

An interesting finding of this study is that although the Complete Dependency−Increasing Group had the poorest preoperative functional status, it caught up with the other two groups by postoperative day 30, with CDS scores approaching the maximum, indicating a state of no dependency. First, the poor baseline status of the Complete Dependency-Increasing Group is mainly attributable to transient and reversible factors, including acute surgical stress, anesthesia effects, and postoperative pain (7). Second, patients received standardized ERAS care, including early mobilization, nutritional support, and routine nursing supervision. These interventions may have disproportionately benefited patients with initially severe dependency, enabling them to regain basic self-care abilities (8). Finally, the Partial Dependency-Stable Group and Very High Dependency-Increasing Group had higher baseline CDS scores, which naturally limited further improvement due to a ceiling effect. Therefore, the convergence of CDS scores toward the maximum reflects both the effectiveness of the standardized ERAS protocol and the transient nature of acute postoperative disability.

Notably, the “Persistent Dependency Group” had significantly lower scores at all five time points compared to the other groups, with all scores remaining below the 70-point threshold for independence. This indicates that these patients continued to require a notable level of care from others even 3 months after surgery. Long-term care dependency can not only hinder the recovery of ADL and psychosocial function but also increase the burden on caregivers (36). In severe cases, it may lead to permanent functional impairment (37). Therefore, patients in this group should be a primary focus for healthcare professionals. Interventions should be initiated starting from the early postoperative phase and extended into post-discharge care through a hospital-community-family integrated model to ensure continuity of support (35).

Compared to the “Partial Dependency-Stable Group,” patients with higher levels of psychological resilience were more likely to belong to the “Very High Dependency-Increasing Group.” Psychological resilience is a positive quality that enables individuals to maintain a healthy mental state when facing trauma or stress, playing a crucial role in mitigating psychological distress (38). Although patients in this group initially exhibited a higher degree of care dependency than those in the Partial Dependency-Stable Group, both groups reached comparable levels of dependency by the T4 and T5 assessments. This convergence may be precisely attributed to their higher psychological resilience, which potentially facilitated a more rapid or effective adaptation and recovery process in the later postoperative phases. However, this finding appears inconsistent with the results of the cross-sectional study by Lenti et al. (39). The discrepancy may stem from methodological differences. Unlike cross-sectional designs which examine variable associations at a single point, our longitudinal approach models trajectories over time. It is plausible that psychological resilience primarily influences the slope of the care dependency trajectory—that is, the rate of change—rather than merely the dependency level at a specific time. These patients likely employ more optimistic attitudes and active coping strategies to confront the initial challenges of their disease, thereby propelling the rehabilitation process, reducing their level of dependency, and ultimately achieving independence. This highlights that the surgical experience constitutes not only a physical but also a significant psychological stressor. Consequently, a patient’s psychological state should be a key focus of holistic postoperative care, alongside the management of physical health. The precise pathways through which psychological resilience shapes care dependency trajectories remain insufficiently understood. Future research is needed to elucidate these mechanisms and their clinical implications.

Compared to the “Partial Dependency-Stable Group,” patients aged >45 and ≤59 years were more likely to be classified into the “Moderate Dependency-Slowly Increasing Group” and the “Complete Dependency-Increasing Group.” First, elderly patients may have reduced preoperative functional ability and emotional state, which could already result in a low level of dependency before surgery (40). Furthermore, advanced age is frequently associated with frailty and multiple comorbidities. The decline in physical functional reserve leads to poorer surgical tolerance, resulting in a higher risk of care dependency in the early postoperative period and often necessitating a prolonged hospital stay for physical recovery (39). Against the backdrop of an aging society and technological advancements in China, an increasing number of elderly individuals are undergoing abdominal surgery. For these older patients, implementing “prehabilitation” can be an effective strategy to enhance physiological reserve and tolerance to surgical stress, thereby reducing the incidence of postoperative complications and the level of care dependency (41). Although the multivariate analysis indicated that age was not a statistically significant factor for classification into the “Persistent Dependency Group,”the univariate analysis revealed that 70% of the patients in this group were aged ≥60 years, compared to 53.91% in the overall sample. This discrepancy may be related to the relatively small sample size of the “Persistent Dependency Group.” Therefore, the possibility that age contributes to a higher degree of dependency cannot be ruled out. Furthermore, after comprehensive consideration, age is also regarded as one of the contributing factors explaining why patients in the “Persistent Dependency Group” did not achieve an independent status even at three months postoperatively (score<70).

Compared to the “Partial Dependency-Stable Group,” patients with lower levels of informational support were more likely to be classified into the “Complete Dependency-Increasing Group” and the “Persistent Dependency Group.” Informational support, which is an important component of social support, addresses cancer patients’ need for more knowledge regarding their disease and treatment. Fulfilling this informational need can effectively reduce patients’ anxiety and depression, thereby lowering their level of illness-related uncertainty (42). Informational support can improve the social functioning of cancer patients, aid in rebuilding their self-efficacy and reinterpreting their role-related responsibilities, thereby facilitating the restoration of personal independence and reducing their level of care dependency. Therefore, nursing staff should gain a comprehensive understanding of the individual informational needs of GC patients, initiating perioperative health education and continuing it into post-discharge home care. For the future, a social network-based intervention program could be established for key postoperative recovery phases. This program could, first, disseminate knowledge about GC through internet-based social platforms. Second, it could provide personalized informational support via patient-nurse “one-to-one”pairing through telephone or online. Finally, establishing dedicated online training groups for family members or caregivers to encourage their participation in family-centered functional training could help improve patients’ daily living abilities, thereby reducing their level of care dependency (43).

Compared to the “Partial Dependency-Stable Group,” patients with a low BMI and low preoperative albumin levels were more likely to be classified into the “Complete Dependency-Increasing Group” and the “Persistent Dependency Group.” BMI is a key indicator reflecting the nutritional status of cancer patients. A low BMI suggests poor nutritional status. GC can induce a systemic inflammatory response, leading to disorders of fat metabolism and protein synthesis, which result in fat loss and decreased skeletal muscle mass. This impairs physical function and surgical tolerance, consequently affecting the incidence of postoperative complications and the patient’s ability to perform daily activities (44). Research (45) has shown that gastric surgery patients with a low BMI experience reduced quality of life in both physical and psychological domains. Furthermore, serum albumin serves as another critical indicator of nutritional status as well as a protein associated with the systemic inflammatory response (46). It plays a pivotal role in regulating plasma colloid osmotic pressure and is a recognized prognostic predictor in patients with GC. Notably, studies indicate that tumor cells release interleukin-6 (IL-6), which suppresses hepatic albumin synthesis via the JAK/STAT3 signaling pathway (47). This process weakens immune functions, such as lymphocyte proliferation and complement activation, thereby increasing the risk of postoperative infections and impairing the recovery of physical function.

Consequently, poor nutritional status adversely affects the rehabilitation process of GC patients. Nursing staff should dynamically monitor the nutritional parameters of these patients and provide timely interventions. These interventions may include, but are not limited to, nutritional health education, oral nutritional supplements (ONS), and preoperative enteral nutrition (48, 49). Such measures are aimed at reducing physical dependency and enhancing overall quality of life.

Compared to the “Partial Dependency-Stable Group,” patients with a later time to first ambulation were more likely to be classified into the “Complete Dependency-Increasing Group” and the “Persistent Dependency Group.” Research indicates that early mobilization promotes postoperative recovery in GC patients. However, due to pain and physical weakness, patient compliance is often poor, and the frequency and intensity of ambulation frequently fail to meet targets. This leads to delayed postoperative recovery and a higher level of care dependency (50). Patients who start ambulation later not only experience a prolonged rehabilitation period and extended hospital stay but may also develop a lack of confidence in their rehabilitation exercises, which can foster withdrawal behaviors and a dependent mindset (51, 52). Therefore, a multidisciplinary team including nursing staff, physical therapists, and occupational therapists should actively encourage and supervise patients in early ambulation. For patients who are unable to walk, active bed-based exercises should be implemented to improve their exercise compliance and subsequently ameliorate their level of care dependency.

## 5 Limitations

Several limitations should be acknowledged. First, we did not separately assess actual ADL using instruments such as the Barthel Index or Functional Independence Measure. Although CDS correlates strongly with these measures, a separate ADL assessment might have provided additional detail on pure functional status. For transparency, the Chinese version of the CDS has been provided as supplementary file C. Second, all participants were recruited from a single tertiary hospital, which may limit the generalizability of the findings. Future studies using a multicenter design are needed to further validate these results. Finally, our study excluded patients who required readmission and severe postoperative complications. While this decision was necessary to ensure the validity of the longitudinal care dependency assessments, it may limit the generalizability of our findings to patients with uncomplicated recovery. Future studies should specifically examine care dependency trajectories in patients with readmission and complications.

## 6 Conclusion

Overall, the care dependency trajectory in patients following laparoscopic radical gastrectomy for GC showed a gradually declining trend. Specifically, LGMM identified four distinct latent classes of trajectories. The analysis further revealed that psychological resilience, age, informational social support, time to first ambulation, BMI, and albumin levels are significant factors influencing these trajectory class memberships. It is crucial for healthcare professionals to identify patients belonging to trajectory classes that deviate from the general recovery pattern and to provide targeted attention and interventions based on these influencing factors.

## Data Availability

The original contributions presented in the study are included in the article/**Supplementary Material**. Further inquiries can be directed to the corresponding author.
