# Supplementary material for: Trajectories of care dependency and predictors following laparoscopic radical gastrectomy for gastric cancer: a longitudinal study
Source: Front Oncol. 2026 Jun 4;16:1799607. doi: 10.3389/fonc.2026.1799607 (PMC13275382; doi:10.3389/fonc.2026.1799607)
Supplement: Supplementary file 1 [file Table1.docx]

STROBE Statement—checklist of items that should be included in reports of observational studies

|  | Item No. | Recommendation | Page  No. | Relevant text from manuscript |
| --- | --- | --- | --- | --- |
| **Title and abstract** | 1 | (*a*) Indicate the study’s design with a commonly used term in the title or the abstract | 1 | This longitudinal observational study included 223 patients who underwent laparoscopic radical gastrectomy for GC. |
|  |  | (*b*) Provide in the abstract an informative and balanced summary of what was done and what was found | 1 | There was heterogeneity in the care dependency trajectories of patients after laparoscopic radical gastrectomy. Nursing staff should effectively identify and pay attention to the trajectory types inconsistent with the overall trend and their influencing factors, and reduce the care dependency level of patients through intervention. |
| Introduction | | | |  |
| Background/rationale | 2 | Explain the scientific background and rationale for the investigation being reported | 2 | This decline consequently creates a need for reliance on others to complete daily activities.With the advancement of Enhanced Recovery After Surgery (ERAS) protocols, the hospital stay for GC patients has been reduced to 6-9 days(7, 8). |
| Objectives | 3 | State specific objectives, including any prespecified hypotheses | 3 | To address this unresolved issue, the present study employed (LGMM, a method that identifies distinct latent subpopulations with different change trajectories(19, 20), to delineate the patterns of care dependency after laparoscopic radical gastrectomy for gastric cancer and to explore their determinants. |
| Methods | | | |  |
| Study design | 4 | Present key elements of study design early in the paper | 3, 7 | This longitudinal descriptive study investigated the experiences of GC patients.Five postoperative follow-up assessments were scheduled: postoperative day 1 (T1), day 3 (T2), day 6 (T3), day 30 (T4), and month 3 (T5). |
| Setting | 5 | Describe the setting, locations, and relevant dates, including periods of recruitment, exposure, follow-up, and data collection | 3 | Participants were recruited from the gastrointestinal surgery department of a tertiary hospital in Guangxi, China between September 2023 and November 2024. |
| Participants | 6 | (*a*) *Cohort study*—Give the eligibility criteria, and the sources and methods of selection of participants. Describe methods of follow-up  *Case-control study*—Give the eligibility criteria, and the sources and methods of case ascertainment and control selection. Give the rationale for the choice of cases and controls  *Cross-sectional study*—Give the eligibility criteria, and the sources and methods of selection of participants | 4 | A convenience sampling method was employed. The inclusion criteria for patients were as follows: (1) Diagnosis consistent with the Chinese Society of Clinical Oncology (CSCO) Gastric Cancer Diagnosis and Treatment Guidelines (2023) (21). (2) Age≥18 years; (3) Successful laparoscopic standard radical gastrectomy for GC (R0 resection); (4) Clear consciousness with adequate comprehension and communication abilities. |
|  |  | (*b*) *Cohort study*—For matched studies, give matching criteria and number of exposed and unexposed  *Case-control study*—For matched studies, give matching criteria and the number of controls per case |  |  |
| Variables | 7 | Clearly define all outcomes, exposures, predictors, potential confounders, and effect modifiers. Give diagnostic criteria, if applicable | 5 | A researcher-designed questionnaire, based on a literature review, was used to collect demographic and clinical data. It included: gender, age, educational level, type of health insurance, marital status, place of residence, primary caregiver, number of comorbid chronic conditions, tumor stage, surgical approach, chemotherapy status and regimen, 6-minute walk distance, NRS2002 score, PG-SGA score, duration of surgery, Body Mass Index (BMI), preoperative albumin level, preoperative total lymphocyte count, t**ime to first ambulation and postoperative le**ngth of hospital stay (LOS). |
| Data sources/ measurement | 8* | For each variable of interest, give sources of data and details of methods of assessment (measurement). Describe comparability of assessment methods if there is more than one group | *7* | The following information was extracted from the hospital‘s electronic medical record system: age, medical history, preoperative laboratory values including albumin, and other relevant clinical variables. ②The PROMIS Social Support, CD-RISC, and IPAQ-SF scales were assessed using self-report questionnaires completed by the patients. ③Nutritional status was assessed by specialized nutrition nurses using the PG‑SGA and NRS2002 before surgery (T0). |
| Bias | 9 | Describe any efforts to address potential sources of bias | 4 | he exclusion criteria were as follows: (1) Other active malignancies; (2) Severe cardiopulmonary/organ dysfunction; (3) Postoperative complications of Clavien-Dindo grades III-V; (4) Eastern Cooperative Oncology Group (ECOG) score < 3 (indicating independence in daily activities prior to surgery, as patients with scores ≥ 3 already have substantial care dependency that could confound the longitudinal analysis); (5) Postop readmission. |
| Study size | 10 | Explain how the study size was arrived at | 4 | Sample size estimation, based on a table for single-group repeated measures(22) (5 measurements, r=0.5, f=0.14, α=0.05, power=0.8) with a 20% attrition buffer, indicated a minimum of 152 patients. Research indicates that when using the Bayesian Information Criterion as the primary consideration for model selection，to ensure model identification accuracy for latent growth modeling(23), the target was set to ≥200. |

Continued on next page

| Quantitative variables | 11 | Explain how quantitative variables were handled in the analyses. If applicable, describe which groupings were chosen and why | 7 | In descriptive analyses (Table 1), these are presented as mean ± standard deviation (SD). In the univariable and multivariable logistic regression models, we first tested these variables in their continuous form. |
| --- | --- | --- | --- | --- |
| Statistical methods | 12 | (*a*) Describe all statistical methods, including those used to control for confounding | 7 | All other statistical analyses were conducted using SPSS software. Continuous variables are presented as mean ± standard deviation and were compared using analysis of variance (ANOVA). Categorical variables are presented as number (percentage) and were compared using the Chi-square test or Fisher's exact test, as appropriate. |
|  |  | (*b*) Describe any methods used to examine subgroups and interactions | 7 | The identified latent class membership was subsequently treated as the dependent variable. Univariate logistic regression analyses were first performed to examine individual associations between predictor variables and class membership. Variables showing a significant association (P<0.05) in the univariate analysis were then entered into a multivariate logistic regression model to identify independent predictors. A two-tailed p-value of less than 0.05 was considered statistically significant. |
|  |  | (*c*) Explain how missing data were addressed |  | - |
|  |  | (*d*) *Cohort study*—If applicable, explain how loss to follow-up was addressed  *Case-control study*—If applicable, explain how matching of cases and controls was addressed  *Cross-sectional study*—If applicable, describe analytical methods taking account of sampling strategy | 4 | A convenience sampling method was employed. |
|  |  | (*e*) Describe any sensitivity analyses |  | - |
| Results | | | | |
| Participants | 13* | (a) Report numbers of individuals at each stage of study—eg numbers potentially eligible, examined for eligibility, confirmed eligible, included in the study, completing follow-up, and analysed | 8 | The mean age of the final cohort (n=206) that completed all follow-ups was 58.57 ± 0.78 years, with a range from 19 to 86 years. |
|  |  | (b) Give reasons for non-participation at each stage | 8 | The overall attrition rate was 7.62% (17/223), with reasons for loss to follow-up including transfer to ICU (n=2), failure to respond to phone calls (n=13), and voluntary withdrawal (n=2). |
|  |  | (c) Consider use of a flow diagram | 8 | The follow-up process is illustrated in Fig. 1. |
| Descriptive data | 14* | (a) Give characteristics of study participants (eg demographic, clinical, social) and information on exposures and potential confounders | Table 1, Table3 | The univariate analysis revealed statistically significant differences among the four trajectory classes in the following variables: age, number of comorbidities, tumor stage, surgical approach, NRS 2002, PG-SGA, 6-minute walking distance, BMI, preoperative albumin level, time to first ambulation, social support score, and psychological resilience score. Detailed results are presented in Table 3. |
|  |  | (b) Indicate number of participants with missing data for each variable of interest |  | - |
|  |  | (c) *Cohort study*—Summarise follow-up time (eg, average and total amount) |  |  |
| Outcome data | 15* | *Cohort study*—Report numbers of outcome events or summary measures over time |  |  |
|  |  | *Case-control study—*Report numbers in each exposure category, or summary measures of exposure |  |  |
|  |  | *Cross-sectional study—*Report numbers of outcome events or summary measures | *11* | The trajectories of the four latent classes are plotted in Figure 2, with mean care dependency scores on the vertical axis and assessment time points on the horizontal axis. Each class was named based on the characteristics of its score trajectory. |
| Main results | 16 | (*a*) Give unadjusted estimates and, if applicable, confounder-adjusted estimates and their precision (eg, 95% confidence interval). Make clear which confounders were adjusted for and why they were included | Table 4 | The logistic regression analysis identified the following factors as significant independent predictors of latent class membership: age, psychological resilience, the informational support dimension of social support, time to first ambulation, BMI, and low preoperative albumin level. The detailed results of the logistic regression are shown in Table 4. |
|  |  | (*b*) Report category boundaries when continuous variables were categorized | Table 3, 15 | NRS 2002 Score: <3 = 0; ≥3 = 1. PG-SGA Score: 0–1 = 1; 2–3 = 2; 4–8 = 3. |
|  |  | (*c*) If relevant, consider translating estimates of relative risk into absolute risk for a meaningful time period |  | - |

Continued on next page

| Other analyses | 17 | Report other analyses done—eg analyses of subgroups and interactions, and sensitivity analyses |  | - |
| --- | --- | --- | --- | --- |
| Discussion | | | | |
| Key results | 18 | Summarise key results with reference to study objectives | 17 | The LGMM analysis delineated four distinct latent classes of care dependency trajectories following laparoscopic radical gastrectomy, all of which demonstrated a dynamic declining trend，with “Partial Dependency-Stable Group” accounting for 5.82%, “Very High Dependency-Increasing Group” accounting for 22.33%, “Complete Dependency-Increasing Group” accounting for 62.13%, “Persistent Dependency Group” accounting for 9.71%. A significant majority of patients (71.8%) were classified into either the “Complete Dependency-Increasing Group” or the “Persistent Dependency Group.” |
| Limitations | 19 | Discuss limitations of the study, taking into account sources of potential bias or imprecision. Discuss both direction and magnitude of any potential bias | 22 | Several limitations should be acknowledged. First, we did not separately assess actual ADL using instruments such as the Barthel Index or Functional Independence Measure. |
| Interpretation | 20 | Give a cautious overall interpretation of results considering objectives, limitations, multiplicity of analyses, results from similar studies, and other relevant evidence | 16-21 | his convergence may be precisely attributed to their higher psychological resilience, which potentially facilitated a more rapid or effective adaptation and recovery process in the later postoperative phases. However, this finding appears inconsistent with the results of the cross-sectional study by Lenti et al.(36). |
| Generalisability | 21 | Discuss the generalisability (external validity) of the study results | 4, 22 | A convenience sampling method was employed. The inclusion criteria for patients were as follows: (1) Diagnosis consistent with the Chinese Society of Clinical Oncology (CSCO) Gastric Cancer Diagnosis and Treatment Guidelines (2023) (21). (2) Age≥18 years; (3) Successful laparoscopic standard radical gastrectomy for GC (R0 resection);  he exclusion criteria were as follows: (1) Other active malignancies; (2) Severe cardiopulmonary/organ dysfunction; (3) Postoperative complications of Clavien-Dindo grades III-V; |
| Other information | |  | | |
| Funding | 22 | Give the source of funding and the role of the funders for the present study and, if applicable, for the original study on which the present article is based | Title page | Title page |

*Give information separately for cases and controls in case-control studies and, if applicable, for exposed and unexposed groups in cohort and cross-sectional studies.

**Note:** An Explanation and Elaboration article discusses each checklist item and gives methodological background and published examples of transparent reporting. The STROBE checklist is best used in conjunction with this article (freely available on the Web sites of PLoS Medicine at http://www.plosmedicine.org/, Annals of Internal Medicine at http://www.annals.org/, and Epidemiology at http://www.epidem.com/). Information on the STROBE Initiative is available at www.strobe-statement.org.
