# Supplementary material for: Trajectories of care dependency and predictors following laparoscopic radical gastrectomy for gastric cancer: a longitudinal study
Source: Front Oncol. 2026 Jun 4;16:1799607. doi: 10.3389/fonc.2026.1799607 (PMC13275382; doi:10.3389/fonc.2026.1799607)
Supplement: Supplementary file 2 [file Table2.docx]

Supplementary File B The scores of CDS for each item at the five time points

|  | T1 | T2 | T3 | T4 | T5 | Change (T5-T1) |
| --- | --- | --- | --- | --- | --- | --- |
| diet | 1.04±0.016 | 1.41±0.053 | 2.45±0.074 | 4.24±0.054 | 4.54±0.044 | 3.50 |
| elimination | 1.68±0.068 | 3.12±0.073 | 3.87±0.066 | 4.97±0.012 | 4.98±0.011 | 3.30 |
| body position | 2.13±0.088 | 3.64±0.076 | 4.44±0.055 | 4.98±0.010 | 4.99±0.007 | 2.86 |
| mobility | 1.57±0.072 | 3.05±0.081 | 3.96±0.056 | 4.94±0.017 | 4.98±0.013 | 3.41 |
| circadian rhythm | 1.92±0.083 | 3.42±0.084 | 4.41±0.059 | 4.86±0.032 | 4.92±0.023 | 3.00 |
| clothing | 1.65±0.075 | 3.09±0.082 | 3.94±0.075 | 4.87±0.029 | 4.94±0.021 | 3.29 |
| temperature | 1.74±0.083 | 3.13±0.086 | 4.18±0.073 | 4.95±0.015 | 4.98±0.010 | 3.24 |
| cleanliness | 1.62±0.081 | 3.23±0.091 | 4.11±0.076 | 4.99±0.007 | 4.98±0.013 | 3.36 |
| risk avoidance | 1.84±0.089 | 3.36±0.084 | 4.18±0.077 | 4.99±0.017 | 4.99±0.008 | 3.15 |
| communication | 2.41±0.110 | 3.61±0.092 | 4.03±0.079 | 4.74±0.042 | 4.79±0.034 | 2.38 |
| socialization | 2.27±0.105 | 3.36±0.096 | 3.87±0.079 | 4.75±0.040 | 4.78±0.036 | 2.51 |
| values and rules | 2.01±0.099 | 3.23±0.097 | 3.79±0.078 | 4.86±0.032 | 4.80±0.034 | 2.79 |
| daily living | 1.59±0.076 | 2.42±0.092 | 3.46±0.082 | 4.26±0.054 | 4.29±0.056 | 2.70 |
| recreational activities | 1.38±0.063 | 1.96±0.089 | 2.32±0.089 | 4.79±0.041 | 4.84±0.032 | 3.46 |
| learning ability | 1.79±0.084 | 3.10±0.087 | 3.80±0.077 | 4.68±0.049 | 4.75±0.041 | 2.96 |
